# The Epidemiology of Lung Metastases

**DOI:** 10.3389/fmed.2021.723396

**Published:** 2021-09-20

**Authors:** Hanbo Chen, Kelsey C. Stoltzfus, Eric J. Lehrer, Samantha R. Horn, Shankar Siva, Daniel M. Trifiletti, Mao-Bin Meng, Vivek Verma, Alexander V. Louie, Nicholas G. Zaorsky

**Affiliations:** ^1^Department of Radiation Oncology, Amsterdam University Medical Centers, Amsterdam, Netherlands; ^2^Department of Radiation Oncology, Penn State Cancer Institute, Hershey, PA, United States; ^3^Department of Public Health Sciences, Penn State Health Milton S. Hershey Medical Center, Hershey, PA, United States; ^4^Department of Radiation Oncology, Icahn School of Medicine at Mount Sinai, New York, NY, United States; ^5^Department of Radiation Oncology, Peter MacCallum Cancer Centre, Melbourne, VIC, Australia; ^6^Department of Radiation Oncology, Mayo Clinic, Jacksonville, FL, United States; ^7^Department of Radiation Oncology, Tianjin Medical University Cancer Institute and Hospital, Tianjin, China; ^8^Department of Radiation Oncology, Allegheny General Hospital, Pittsburgh, PA, United States; ^9^Department of Radiation Oncology, Sunnybrook Health Sciences Centre, Toronto, ON, Canada

**Keywords:** lung cancer, metastases, cancer, epidemiology, oncology

## Abstract

**Introduction:** Lung metastasis is usually associated with poor outcomes in cancer patients. This study was performed to characterize and analyze the population of patients with *de novo* (synchronous) lung metastases using the Surveillance, Epidemiology and End Results (SEER) database.

**Materials and Methods:** Baseline characteristics of lung metastasis patients were obtained from SEER case listings. Incidence rates and counts of synchronous lung metastasis were also obtained using the SEER^*^Stat software. Survival outcomes were analyzed using univariate and multivariable Cox regressions, controlling for confounders. An alpha threshold of 0.05 was used for statistical significance and *p*-values were subject to correction for multiple comparisons.

**Results:** The age-adjusted incidence rate of synchronous lung metastasis was 17.92 per 100,000 between 2010 and 2015. Synchronous lung metastases most commonly arose from primary lung cancers, colorectal cancers, kidney cancers, pancreatic cancers and breast cancers. During this time period, 4% of all cancer cases presented with synchronous lung metastasis. The percentage of patients presenting with synchronous lung metastasis ranged from 0.5% of all prostate cancers to 13% of all primary lung cancers. The percentage of all cancer cases presenting with synchronous lung metastasis increased over time. *De novo* metastatic patients with lung metastases had worse overall survival [hazard ratio = 1.22 (1.21–1.23), *p* < 0.001] compared to those with only extrapulmonary metastases, controlling for potential confounders.

**Conclusions:** Synchronous lung metastasis occurs frequently and is an independent predictors of poor patient outcomes. As treatment for lung metastases becomes more complicated, patients with synchronous lung metastasis represent a high-risk population.

## Introduction

The diagnosis of metastasis heralds death in most cancer patients. Lung metastases are frequently observed across many primary cancer sites and are commonly considered to confer poor prognosis (1–7). The healthcare impact of lung metastases, whose treatment grows increasingly nuanced (8–10), is also a significant public health concern.

Despite the pervasiveness of lung metastasis as a cancer phenomenon, its epidemiology has not yet been systematically described at a population level. Current research mainly focuses on the biological mechanisms and treatment of lung metastasis across different primary sites (11–16). The existing epidemiological studies of lung metastasis have either not been able to provide patient-level data (17, 18) or have focused on patients from a single primary site, limiting generalizability (2, 19–22).

Among metastases, synchronous metastases that appear at, or close to, the time of presentation appear to be a distinct entity that may be associated with inferior outcomes in certain primary sites (23–25). As research into the treatment of metastases accelerates, it is increasingly important to provide a description of the disease frequency and general outcomes of patients with *de novo*, synchronous lung metastasis across all primary sites. The population-level Surveillance, Epidemiology and End Results (SEER) database is a powerful source of data to address these questions.

This epidemiological study had three objectives. Firstly, we sought to describe the population of patients with *de novo* (synchronous) lung metastases and compare it to the overall cancer population. Secondly, we wished to investigate the trend in the frequency of synchronous lung metastases across time and age at diagnosis for all primary sites. Lastly, we aimed to compare the overall survival of all *de novo* metastatic patients stratified by the presence of lung metastases across all primary sites.

## Materials and Methods

### Data Acquisition

The SEER database covers ~28% of the population of the United States (US) as of 2017 (26). The SEER^*^Stat software (v8.3.5) was used to query the SEER 18-registry research database (November 2017 submission) (27). All relevant ethics regulations are observed. This study was exempt from Institutional Review Board review due to the usage of a publicly available, anonymous database. For incidence rates, age-adjusted incidence rates standardized to the 2000 US census population were obtained for patients with *de novo* (synchronous) lung metastases from 2010 to 2015 using the SEER^*^Stat software. *De novo* (synchronous) lung metastases were defined as those found at the time as the primary cancer diagnosis and contributed to the initial staging of the primary cancer. This is in contrast to recurrent (metachronous) lung metastases, which arise after the initial diagnosis of the primary cancer. The SEER^*^Stat software was also used to calculate the annual percent change in the incidence rates of synchronous lung metastasis *via* weighted linear regression methods (28).

For the survival analysis, case listings were obtained using the SEER^*^Stat software for cases with and without synchronous lung metastasis from 2010 to 2015. Cases where the lung metastasis status was unknown, where the M-Stage status was unknown or where the survival duration was missing were excluded.

### Statistical Analysis

All statistical analysis was carried out in the R statistical platform (v3.6.1 x64). An alpha threshold of 0.05 was used for statistical significance. All *P*-values reported were two-sided. Comparisons of baseline statistics were performed using Student's *t*-tests for continuous variables, chi-squared tests for categorical variables and log-rank tests for time to event variables, where appropriate. The Cochran-Armitage test was used for the analysis of overall and site-specific time trends. Results obtained from multiple tests on the same patient population, such as survival analysis stratified by primary site, were subject to Holm's correction for multiple testing (29, 30).

For survival analysis, the population used was all cases with *de novo* metastatic cancer (M1+ by the American Joint Committee on Cancer 7th edition definition) in the SEER database from 2010 to 2015. Overall survival functions were estimated by the Kaplan-Meier method. Univariate Cox proportional hazards regression were used to compare the hazards of death for metastatic cases with lung metastasis vs. those with only extrapulmonary metastases. The potential confounders of age, sex, race, year of diagnosis, T-stage, nodal status and the presence of bone, brain or liver metastasis were adjusted for in multiple Cox regression models. Reported hazard ratios are followed by their 95% confidence intervals in brackets. Effect modification by age, sex, and race was investigated by the addition of interaction terms to the multiple Cox regression models. Records with missing values consisted <0.1% of the survival dataset and were therefore omitted in statistical analysis.

## Results

### Incidence of Synchronous Lung Metastasis

Between 2010 and 2015, a total of 100,751 cases of synchronous lung metastasis in the were captured by the SEER registries. Baseline characteristics of these patients are shown in Table 1. Compared to other cancer patients without synchronous lung metastasis, patients with synchronous lung metastasis were more likely to be older, male, non-white, and had more advanced T- and N-stage at diagnosis.

**Table 1:** Summary of baseline characteristics for all cancer cases with and without synchronous lung metastasis in the United States from 2010 to 2015.

	**Synchronous lung metastasis (***N*** = 100,751)**	**No synchronous lung metastasis (***N*** = 1,996,258)**	***P*** **-value**
Age, mean (standard deviation)	66.8 (14.3)	64.1 (14.5)	<0.001
**Sex**			
Female	48,819 (48.5%)	1,010,788 (50.6%)	<0.001
Male	51,932 (51.5%)	125,649 (49.4%)	
**Year of diagnosis**			
2010	15,202 (15.1%)	325,629 (16.3%)	<0.001
2011	15.934 (15.8%)	329,677 (16.5%)	
2012	16,819 (16.7%)	329,712 (16.5%)	
2013	17,331 (17.2%)	332,026 (16.6%)	
2014	17,690 (17.6%)	336,711 (16.9%)	
2015	17,775 (17.6%)	342,503 (17.2%)	
**Presence of bone, brain or liver metastasis at diagnosis**			
Yes	54,937 (54.5%)	164,491 (8.2%)	<0.001
No	45,814 (45.5%)	1,831,767 (91.8%)	
**Race**			
American Indian/Alaska Native	792 (0.8%)	11,573 (0.6%)	<0.001
Asian or Pacific Islander	7,953 (7.9%)	132,326 (6.6%)	
Black	13,202 (13.1%)	213,513 (10.7%)	
White	78,589 (78.0%)	1,611,906 (80.7%)	
Unknown	215 (0.2%)	26,940 (1.3%)	
**T-Stage**			
T0	1,671 (1.7%)	6,045 (0.3%)	<0.001
T1	7,979 (7.9%)	808,117 (40.5%)	
T2	13,431 (13.3%)	473,803 (23.7%)	
T3	24,682 (24.5%)	320,577 (16.1%)	
T4	29,894 (29.7%)	141,626 (7.1%)	
TX	19,045 (18.9%)	124,536 (6.2%)	
Other T	0 (0%)	62,460 (3.1%)	
Missing	4,409 (4.0%)	59,094 (3.0%)	
**N-Stage**			
N0	30,912 (30.7%)	1,411,811 (70.7%)	<0.001
N+	53,580 (53.2%)	464,279 (23.3%)	
NX	12,103 (12.0%)	60,992 (3.1%)	
Missing	4,156 (4.1%)	59,176 (3.0%)	
**M-Stage**			
M1	96,693 (96.0%)	237,074 (11.9%)	
Other M-Stage	12 (<0.1%)	1,700,109 (85.2%)	
Missing	4,046 (4.0%)	59,075 (3.0%)	
**Survival duration missing**			
No	100,584 (99.8%)	1,995,011 (99.9%)	<0.001
Yes	167 (0.2%)	1,247 (<0.1%)	
Median follow-up duration (months, 95% confidence interval)	33 (32–33)	34 (34–34)	0.01

*P-values represent results from t-tests, chi-squared tests or log-rank tests for continuous, categorical and time to event variables, respectively, where appropriate. T-Stage, N-Stage, and M-Stage represent those from the American Joint Committee on Cancer 7th Edition Staging Manual*.

**Figure 1:**
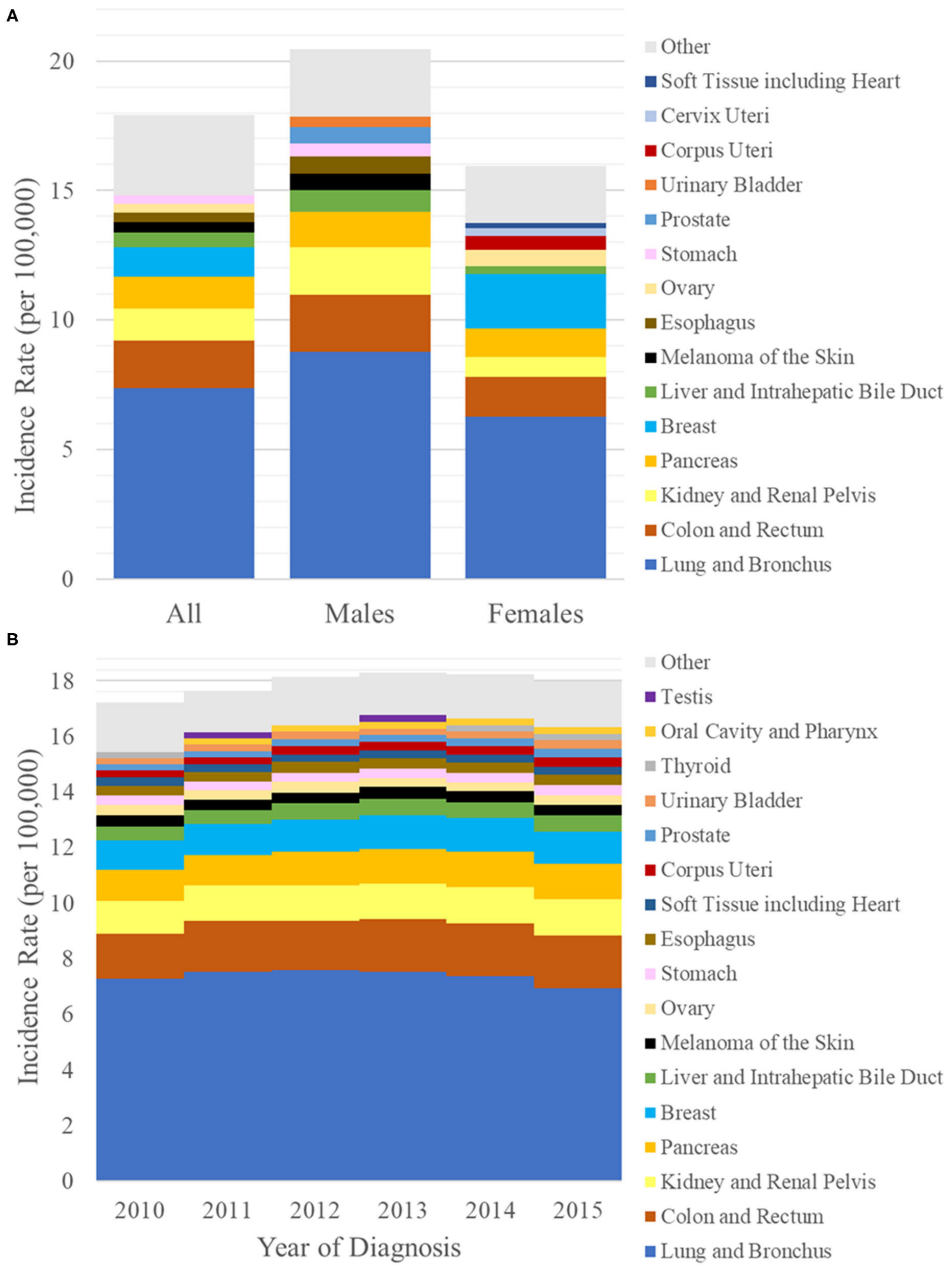
**(A)** Bar graph of the incidence rates per 100,000 of synchronous lung metastasis (y-axis) from 2010 to 2015 in the general population (left) and in either sex (males: middle, females: right). **(B)** Bar graph of the incidence rates per 100,000 of synchronous lung metastasis (y-axis) vs. year of diagnosis (x-axis) from 2010 to 2015. Each bar is broken down by primary sites of origin, which are indicated by different colors.

The age-adjusted incidence rate of *de novo* lung metastasis between 2010 and 2015 was 17.92 cases per 100,000. The incidence rate was 20.46 in males and 15.95 in females (Figure 1). As a reference, the age-adjusted incidence rate of all cancers between 2010 and 2015 was 442.0 cases per 100,000 (males: 489.3; females: 410.0). Therefore, the percentage of all incident cancer cases with synchronous lung metastasis was 4.04% (males: 4.13%, females: 3.95%). In comparison, the percentage of all incident cancer cases that were primary lung cancers was 12.4% (males: 12.8%, females: 12.0%).

The primary sites that contributed the most to the incidence rate of synchronous lung metastasis were lung and bronchus at 7.37 per 100,000 (primary site for 41% of all synchronous lung metastasis), colon and rectum at 1.83 per 100,000 (10%), kidney and renal pelvis at 1.26 per 100,000 (7%), pancreas at 1.21 per 100,000 (7%), and breast at 1.15 per 100,000 (6%) (Figure 1A). Prominent primary sites that were most likely to be metastatic to lung on presentation were lung and bronchus (13%), bile duct (11%), pancreas (10%), esophagus (10%), and soft tissues (8%). Prominent epithelial primary sites that were least likely to be metastatic to lung on presentation were prostate (0.5%), vulva (1%), bladder (1%), thyroid (1%), and breast (2%). The breakdown of most common primary sites differed by sex. A complete list of the incidence rates of synchronous lung metastasis used to generate Figure 1A can be found in Supplementary Table 1.

### Time Trend in Synchronous Lung Metastasis

The incidence rates of synchronous lung metastasis were 17.20, 17.64, 18.15, 18.30, 18.23, and 17.96 per 100,000, respectively, from 2010 to 2015 (Figure 1B). The proportion of cancer cases presenting with lung metastases were 3.74, 3.87, 4.08, 4.17, 4.20, and 4.17% (Table 2), respectively from 2010 to 2015, showing a trend of increase (Cochran-Armitage test *P* < 0.001).

**Table 2:** Time trend of the percent of all incidence cancer cases presenting with synchronous lung metastasis in the United States from 2010 to 2015, stratified by primary site of origin.

**Site**	**Percent of all incident cancer cases presenting with synchronous lung metastasis (%)**						***P*** **-value**	**Adjusted ***P***-value**	**Trend**
	**2010**	**2011**	**2012**	**2013**	**2014**	**2015**			
All sites	3.74	3.87	4.08	4.17	4.20	4.17	<0.001	<0.001*	Increasing
Anus, anal canal and anorectum	1.72	1.88	2.05	1.65	2.27	2.20	0.250	1.000	
Bones and joints	11.31	10.90	11.18	10.10	8.52	11.83	0.522	1.000	
Brain	0.04	0.09	0.09	0.09	0.11	0.07	0.536	1.000	
Breast	1.62	1.67	1.72	1.80	1.82	1.70	0.036	0.720	
Cervix uteri	3.32	3.73	4.44	4.02	4.31	4.90	0.001	0.027*	Increasing
Colon and rectum	3.91	4.50	4.52	4.96	4.86	5.04	<0.001	<0.001*	Increasing
Corpus uteri	1.92	1.98	2.09	2.31	2.21	2.27	0.014	0.312	
Esophagus	8.13	8.52	9.03	9.04	8.37	9.81	0.033	0.693	
Gallbladder	3.47	4.21	5.68	5.00	4.76	5.33	0.068	1.000	
Kidney and renal pelvis	7.62	8.14	8.09	8.02	8.29	8.26	0.062	1.000	
Larynx	1.64	2.32	1.86	2.41	2.48	1.73	0.481	1.000	
Liver and intrahepatic bile duct	6.00	5.95	6.54	6.54	6.06	6.38	0.357	1.000	
Lung and bronchus	12.41	13.28	13.57	13.74	13.68	13.30	<0.001	<0.001*	Increasing
Melanoma of the skin	1.83	1.71	1.78	1.92	1.70	1.64	0.224	1.000	
Mesothelioma	6.14	6.24	5.54	7.60	4.56	6.39	0.811	1.000	
Oral cavity and pharynx	1.73	1.98	2.11	2.12	2.15	2.18	0.018	0.390	
Other biliary	5.32	5.65	5.48	5.49	6.84	6.13	0.107	1.000	
Ovary	5.66	5.32	5.94	5.13	5.36	5.78	0.963	1.000	
Pancreas	8.98	8.79	9.69	9.84	9.96	9.94	<0.001	0.006*	Increasing
Peritoneum, omentum and mesentery	6.09	5.12	7.40	5.66	8.06	7.16	0.168	1.000	
Prostate	0.34	0.31	0.49	0.50	0.58	0.64	<0.001	<0.001*	Increasing
Small intestine	2.79	2.32	1.61	1.95	2.65	2.37	0.814	1.000	
Soft tissue including heart	8.77	8.13	8.03	7.84	8.25	8.26	0.586	1.000	
Stomach	4.70	4.25	4.60	4.74	4.94	4.92	0.122	1.000	
Testis	6.47	7.62	6.55	8.14	6.57	7.08	0.740	1.000	
Thyroid	1.47	1.25	1.24	1.32	1.43	1.50	0.321	1.000	
Ureter	3.37	4.06	3.64	3.25	3.54	4.29	0.693	1.000	
Urinary bladder	1.07	1.21	1.29	1.21	1.40	1.66	<0.001	<0.001*	Increasing
Vulva	1.67	1.61	1.20	1.01	0.97	1.07	0.053	0.998	

*P-value represents those obtained from Cochran-Armitage tests and the adjusted P-values were corrected for multiple-testing. The direction of the change over time was represented in the Trend column where the test for trend was statistically significant*.

* *Statistically significant*.

The sites where the increase in proportion of incident cases presenting with lung metastases were the greatest were cervix uteri (*P* = 0.027), colon and rectum (*P* < 0.001), lung and bronchus (*P* < 0.001), pancreas (*P* = 0.006), prostate (*P* < 0.001), and urinary bladder (*P* < 0.001), as shown in Table 2. As an exploratory analysis, the annualized percent change in the absolute incidence rates of synchronous lung metastasis from 2010 to 2015 was also obtained from SEER (Supplementary Table 2). No significant change in the absolute number of synchronous lung metastasis cases was observed.

**Figure 2:**
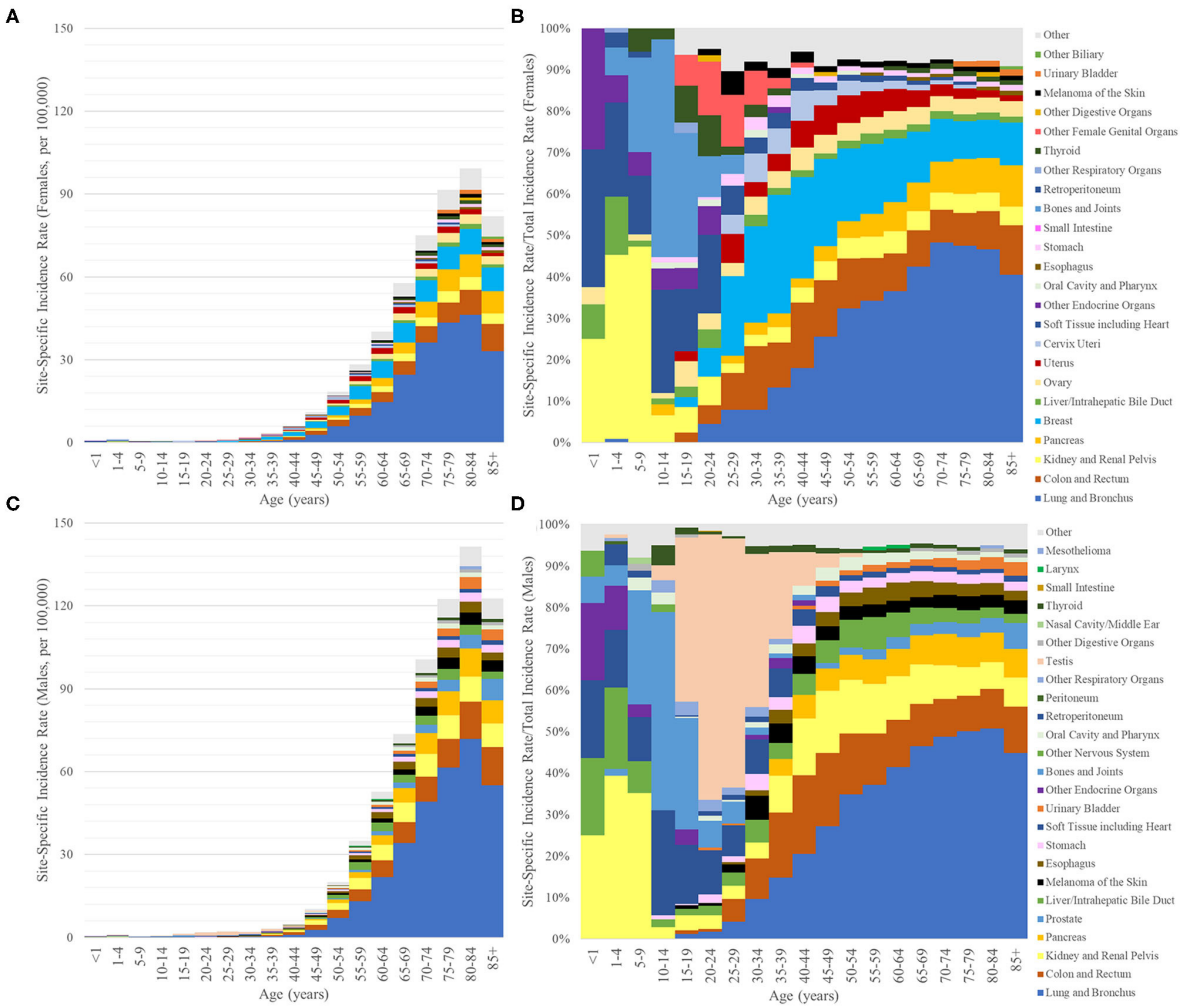
Bar graphs of the incidence rates per 100,000 of synchronous lung metastasis (y-axis) vs. age at diagnosis (x-axis, 5-year bins) broken down by primary sites of origin, which are indicated by different colors (only the top 10 for ages <15 and top 15 for ages >15). **(A,B)** Female. **(C,D)** Male. **(A,C)** y-axis values represent absolute incidence rates. **(B,D)** y-axis values represent the percentage of synchronous lung metastasis from one primary site relative to the overall incidence of synchronous lung metastasis from all primary sites for that age group.

The incidence rate of synchronous lung metastasis increased with age and reached a maximum between ages 80–84 for the entire population (116.4 per 100,000) as well as for both genders (men: 141.4, women: 99.3) (Figure 2). The primary sites that contributed the most to synchronous lung metastasis changed across different age groups. In those under 10, cancers starting in the kidney and soft tissues dominated in females, while cancers starting in the kidney, soft tissues, and liver/biliary system dominated in males. In those between 10 and 20, cancers starting in the bone and soft tissues were major contributors to synchronous lung metastasis in both sexes. In males, the dominance of testicular cancers in contributing to synchronous lung metastasis was apparent from 15 to 40. However, the overall incidence of synchronous lung metastasis remained very low for those under 40 (1.39 per 100,000 for all cases <40). In those above 40 where synchronous lung metastasis was much more common (39.8 per 100,000 for all cases >40), the distribution of primary sites resembled those reported in the prior section. A complete list of the incidence rates of synchronous lung metastasis used to generate Figure 2 can be found in Supplementary Table 3.

### Survival Analysis

A total of 96,535 cases with *de novo* lung metastasis and 236,875 cases with *de novo* metastatic cancer but no lung metastases were included in the survival analysis. A detailed breakdown of the baseline characteristics of the cases included in the survival analysis can be found in Supplementary Table 4.

**Figure 3:**
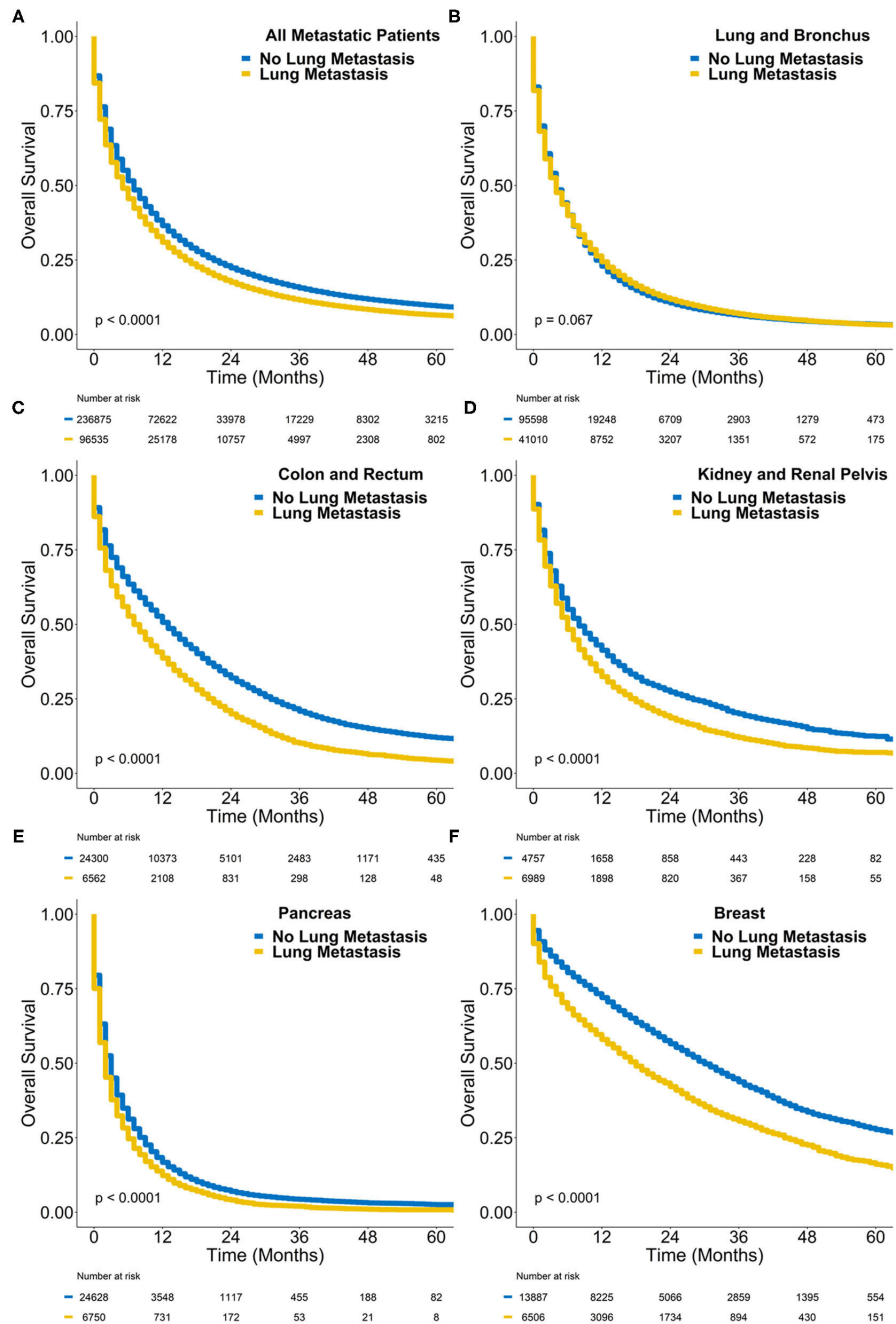
Kaplan-Meier survival plot of overall survival probability (y-axis) vs. time in months (x-axis) comparing *de novo* metastatic cases with synchronous lung metastasis (yellow) to those without (blue) in **(A)** all cases, **(B)** lung cancer cases, **(C)** colorectal cancer cases, **(D)** kidney cancer cases, **(E)** pancreatic cancer cases, and **(F)** breast cancer cases. The *P*-value represents the results of a log-rank test.

At a median follow-up of 33 months, the median survival of all metastatic cases with synchronous lung metastasis was 5 months, and the 2-year overall survival was 17.4% (Figure 3). In comparison, the median survival of all *de novo* metastatic cases with only extrapulmonary metastases was 7 months and the 2-year overall survival was 22.3% (log-rank *P* < 0.0001). Table 3 contains the median overall survival and 2-year overall survival of all primary sites with and without synchronous lung metastasis. The Kaplan-Meier curves comparing the survival of those with synchronous lung metastasis vs. those without for the sites with the highest incidences of synchronous lung metastases are also plotted in Figure 3.

**Table 3:** Results of univariate and multivariable cox regression comparing the hazards of death of *de novo* metastatic patients with lung metastasis vs. those without lung metastasis.

**Univariate Analysis**	**HR**	**95% CI**	***P*** **-value**
Lung metastasis vs. no lung metastasis	**1.18**	**[1.17–1.19]**	** <0.001**
**Multivariable Analysis**			
Lung metastasis vs. no lung metastasis	**1.22**	**[1.21–1.23]**	** <0.001**
Male vs female sex	1.12	[0.89–1.11]	<0.001
Year of diagnosis (per each year increase)	0.98	[0.98–0.98]	<0.001
Age (per each year increase)	1.02	[1.02–1.02]	<0.001
**T-Stage**			
T0 vs. T1	1.13	[1.10–1.17]	<0.001
T2 vs. T1	1.19	[1.17–1.21]	<0.001
T3 vs. T1	1.10	[1.09–1.12]	<0.001
T4 vs. T1	1.29	[1.28–1.31]	<0.001
TX vs. T1	1.48	[1.45–1.50]	<0.001
**N-Stage**			
N+ vs. N0	1.17	[1.16–1.19]	<0.001
NX vs. N0	1.17	[1.16–1.18]	<0.001
**Race**			
American Indian/Alaska Native vs. White	1.03	[0.98–1.08]	0.193
Asian/Pacific Islander vs. White	0.86	[0.85–0.88]	<0.001
Black vs. White	1.07	[1.06–1.08]	<0.001
Unknown vs. White	0.50	[0.44–0.55]	<0.001
Co-existing bone/brain/liver metastasis vs. no bone/brain/liver metastasis	1.25	[1.24–1.26]	<0.001

*HR, hazard ratio; CI, confidence interval*.

On univariate Cox regression, the presence of synchronous lung metastasis was associated with reduced overall survival compared to patients with only extrapulmonary metastases [hazard ratio (HR) = 1.18, 95% confidence interval (1.17–1.19), *P* < 0.001]. On multiple Cox regression controlling for age, sex, race, year of diagnosis, T-stage, nodal status, and the co-existing presence of bone, brain or liver metastasis at diagnosis, the presence of synchronous lung metastasis was still associated with poorer overall survival [HR = 1.22 (1.21–1.23), *P* < 0.001], as shown in Table 4.

**Table 4:** Sample size, median survival, 2-year survival and adjusted hazard ratios for death comparing *de novo* metastatic cases with and without lung metastasis, stratified by primary site of origin.

**Site**	***N***	**Median follow-up (m)**	**Lung metastasis**		**No lung metastasis**		**HR**	**95% CI**	***P*** **-value**	**Adjusted ***P***-value**
			**MS (m)**	**2-year OS (%)**	**MS (m)**	**2-year OS (%)**				
All sites	333,410	33	5	17.5	7	22.3	1.22	[1.21–1.23]	<0.001	<0.001*
Anus, anal canal and anorectum	706	36	12	27.5	18	38.6	1.52	[1.22–1.88]	<0.001	0.002*
Biliary tract	5,836	31	3	4.5	4	7.9	1.25	[1.17–1.34]	<0.001	<0.001*
Bone and soft tissue	3,290	33	11	29.3	12	34.4	1.29	[1.18–1.42]	<0.001	<0.001*
Breast	20,393	33	18	42.1	30	56.5	1.46	[1.40–1.52]	<0.001	<0.001*
Cervix uteri	2,612	30	6	17.7	14	33.6	1.47	[1.33–1.63]	<0.001	<0.001*
Colon	30,862	34	7	19.9	13	32.0	1.20	[1.16–1.24]	<0.001	<0.001*
Esophagus	6,859	36	4	6.8	6	9.5	1.26	[1.19–1.33]	<0.001	<0.001*
Hypopharynx	273	41	8	12.7	6	11.4	1.04	[0.79–1.38]	0.779	1.000
Kidney and renal pelvis	11,746	33	6	18.6	8	27.3	1.45	[1.39–1.52]	<0.001	<0.001*
Larynx	564	34	7	14.3	10	25.1	1.71	[1.38–2.12]	<0.001	<0.001*
Liver	5,051	31	1	4.6	3	6.5	1.46	[1.37–1.55]	<0.001	<0.001*
Lung and bronchus	136,608	34	4	11.8	4	10.6	1.00	[0.99–1.02]	0.488	1.000
Melanoma of the skin	4,496	30	6	18.9	12	35.0	1.47	[1.36–1.58]	<0.001	<0.001*
Mesothelioma	970	34	4	7.5	5	16.8	1.10	[0.94–1.30]	0.237	1.000
Nasopharynx	368	32	14	31.8	17	38.0	1.43	[1.06–1.92]	0.019	0.167
Oral cavity excl. tongue	338	29	7	22.6	8	19.9	1.23	[0.92–1.63]	0.157	0.942
Oropharynx	170	37	5	7.1	11	14.1	1.85	[1.26–2.73]	0.002	0.019*
Ovary	8,631	34	12	32.9	19	43.3	1.27	[1.19–1.35]	<0.001	<0.001*
Pancreas	31,378	32	2	4.1	3	7.0	1.22	[1.19–1.26]	<0.001	<0.001*
Penis	77	21	6	10.0	10	29.1	1.70	[0.90–3.20]	0.102	0.714
Peritoneum, omentum and mesentery	1,002	36	22	46.9	26	51.0	1.15	[0.93–1.41]	0.207	1.000
Prostate	17,241	31	18	40.6	26	52.2	1.36	[1.26–1.46]	<0.001	<0.001*
Rectosigmoid junction	3,500	35	12	27.3	18	39.5	1.18	[1.08–1.30]	<0.001	0.003*
Rectum	7,685	33	14	30.8	18	39.9	1.24	[1.17–1.32]	<0.001	<0.001*
Retroperitoneum	318	36	9	25.5	20	45.5	1.57	[1.18–2.08]	0.002	0.019*
Salivary gland	444	32	11	29.4	12	25.6	1.29	[1.00–1.66]	0.046	0.371
Small intestine	2,987	32	6	21.3	32	54.9	1.63	[1.40–1.90]	<0.001	<0.001*
Stomach	12,207	32	3	6.8	5	12.3	1.26	[1.20–1.33]	<0.001	<0.001*
Testis	1,697	33	NR	70.8	NR	83.9	2.14	[1.69–2.70]	<0.001	<0.001*
Thyroid	1,702	32	10	39.6	69	63.6	1.63	[1.39–1.92]	<0.001	<0.001*
Tongue	581	28	7	22.9	11	27.8	1.56	[1.26–1.93]	<0.001	0.001*
Tonsil	383	30	10	21.2	15	40.4	1.77	[1.36–2.31]	<0.001	<0.001*
Ureter	283	39	5	3.2	6	11.5	1.15	[0.88–1.51]	0.314	1.000
Urinary bladder	4,002	35	4	7.6	5	11.0	1.24	[1.15–1.33]	<0.001	<0.001*
Uterus	6,000	32	7	19.7	15	35.1	1.52	[1.42–1.62]	<0.001	<0.001*
Vulva and vagina	500	29	4	16.5	12	31.3	2.32	[1.86–1.91]	<0.001	<0.001*

*P-values represent those from multiple Cox regression models and the adjusted P-values were corrected for multiple testing*.

* *Statistically significant*.

*MS, median survival; HR, hazard ratio; CI, confidence interval; NR, not reached*.

**Figure 4:**
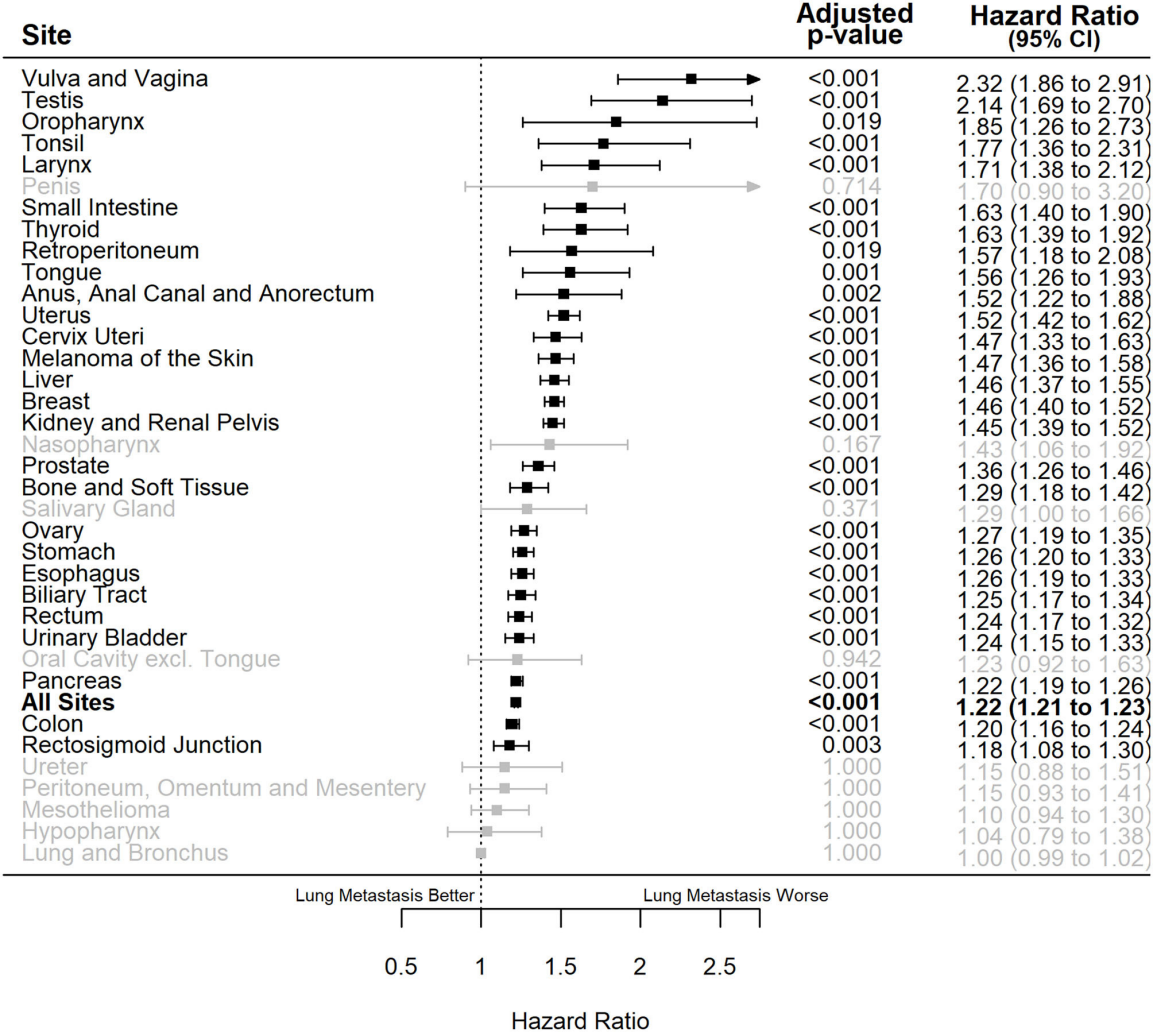
Forest plot of adjusted hazard ratios of death (x-axis) for *de novo* metastatic cases with lung metastasis vs. those with only extrapulmonary metastases for different primary sites of origin (y-axis). Bold: the overall effect estimate for all sites, black: statistically significant individual primary sites, gray: non-statistically significant individual primary sites. Adjusted *P*-values represent those corrected for multiple testing. CI, confidence interval.

Multiple Cox regressions were also performed for all primary sites (Table 3). The primary sites where synchronous lung metastasis had the greatest negative impact on the overall survival of metastatic patients, considering all other influencing factors, were vulva and vagina [HR = 2.32 (1.86–2.91), *P* < 0.001], testis [HR = 2.14 (1.69–2.70), *P* < 0.001], oropharynx [excluding tongue and tonsil, HR = 1.85 (1.26–2.73), *P* = 0.019], tonsil [HR = 1.77 (1.36–2.31), *P* < 0.001], and larynx [HR = 1.71 (1.38–2.12), *P* < 0.001]. Interestingly, synchronous lung metastasis did not impact the overall survival of metastatic lung and bronchus cancer cases when other confounding factors were controlled (Figure 4).

The negative effect of synchronous lung metastasis on the overall survival of *de novo* metastatic cases was especially exacerbated in males [HR = 1.29 (1.27–1.30), *P* < 0.001] compared to females [HR = 1.15 (1.14–1.17), *P* < 0.001] (*P* for interaction < 0.001). The effect of synchronous lung metastasis on survival was also slightly exacerbated in younger (age <65) cases [HR = 1.24 (1.22–1.26), *P* < 0.001] compared to elderly (age >65) cases [HR = 1.20 (1.18–1.21), *P* < 0.001] (*P* for interaction < 0.001). There was no effect modification by race.

## Discussion

This is the first known report of the general epidemiology of synchronous lung metastasis. In summary, 1 in 25 cancer cases in the SEER population presented with synchronous lung metastasis from 2010 to 2015, representing a significant public health concern. Concerningly, the proportion of cancer cases presenting with synchronous lung metastasis has been increasing during this time, led by increases in high-incidence primary sites such as colorectal cancers, lung cancers and prostate cancers. On survival analysis, *de novo* metastatic patients with lung metastasis had lower overall survival compared to those with only extrapulmonary metastases.

In comparison to previously published results, Mitry et al. reported that 2.1% of colorectal cancers presented with synchronous lung metastasis in a cohort of 6,996 French colorectal cancer patients between 1976 and 2005 (2). This is somewhat lower than the SEER data of 3.9–5% between 2010 and 2015 (Table 2). However, Mitry et al. also reported a significant increase in the proportion of metastatic colorectal patients presenting with synchronous lung metastasis over time. Similarly, van der Geest et al. also reported an increase in the proportion of all colorectal patients presenting with lung metastasis, going from 1.7 to 5% between 1996 and 2011 (21). This time trend observed both in our study and previous studies likely represented advancements in imaging technologies with the introduction of wide-spread computed tomography (CT) and later positron emission tomography (PET) in recent years (31). However, it is uncertain whether there are other potential drivers for the increase in the proportion of cases with synchronous lung metastasis. Specifically, it is possible that the increase of synchronous lung metastasis in prostate cancer arose more from the decreasing popularity of prostate-specific antigen screening, leading to more advanced disease at presentation (32, 33).

Interestingly, the finding that synchronous lung metastasis did not have an effect on the survival of *de novo* metastatic lung cancer cases would suggest that the overall survival of Stage IV lung cancer cases depended much more on other factors such as age, sex, T/N-Stage, and the presence of other sites of metastases. It is useful to note that synchronous lung metastasis in the SEER database meant solid metastases in the lung only, excluding pleural metastases or pleural effusions (34). It is possible that synchronous lung metastases that develop from lung cancers were molecularly less aggressive due to the similarities of the primary and metastatic host microenvironments (35). It could also be that a small proportion of these metastatic cases represented occult, synchronous, early-stage primary cancers (36) that had a better prognosis. To offer further insight into the effect of synchronous lung metastasis on the survival of metastatic lung cancer patients, we re-analyzed lung cancer cases looking for effect modification by small cell histology in multiple regression models. It appeared that in metastatic non-small cell lung cancer, synchronous lung metastasis had no impact on the overall disease trajectory of the patient [HR = 0.99 (0.98–1.01)], whereas there was a statistically significant relationship between synchronous lung metastasis and survival in small cell lung cancers [HR = 1.10 (1.06–1.14)].

Our study was limited by several factors. The SEER database does not collect data on subsequent (i.e., metachronous) metastases. There have been instances of attempting to use the linked US Medicare database to obtain linked information on metachronous metastases, but this approach is likely limited by the potential of under- or mis-identifying metachronous cases (37). The SEER database also lacks the total number of metastases at diagnosis, precluding an analysis of oligometastatic vs. non-oligometastatic disease at diagnosis. Baseline performance status is also not captured in the SEER database. As a result of these limitations, we have not compared the effect of treatment options such as surgery, radiotherapy, or systemic therapy on the survival of patients with synchronous lung metastasis due to the inability to adjust for the number of metastases and performance status.

## Conclusion

In this study, we reported the epidemiology and survival impact of synchronous lung metastasis. From a public health perspective, reducing the incidence of lung, colorectal, kidney, pancreatic and breast cancers would have the greatest effect on reducing the incidence of synchronous lung metastasis. From a clinical perspective, synchronous lung metastasis had the greatest impact on the prognosis of vulvar/vaginal, testicular, oropharyngeal wall, tonsillar, and laryngeal cancers, necessitating extra care in the management of these patients.

## Data Availability Statement

The original contributions generated for the study are included in the article/Supplementary Material, further inquiries can be directed to the corresponding author/s.

## Author Contributions

EL, KS, SS, DT, VV, AL, and NZ contributed conceptualization of the study. HC, EL, KS, SH, and NZ performed the statistical analyses. All authors contributed to manuscript revision, read, and approved the final submitted version.

## Funding

NZ is supported by startup funding from Penn State Cancer Institute and Penn State College of Medicine. NZ is supported by the National Institutes of Health Grant LRP 1 L30 CA231572-01. NZ is supported by the American Cancer Society - Tri State CEOs Against Cancer Clinician Scientist Development Grant, CSDG-20-013-01-CCE. NZ received remuneration from Springer Nature for his textbook, Absolute Clinical Radiation Oncology Review.

## Conflict of Interest

NZ received personal fees from Springer Nature Inc. and Weatherby Healthcare, unrelated to the submitted work. DT reports clinical trial research support from Novocure, and publishing fees from Springer Nature Inc. for projects unrelated to the submitted work. AL has received honoraria from Varian Medical Systems Inc. and AstraZeneca, unrelated to the current work. The remaining authors declare that the research was conducted in the absence of any commercial or financial relationships that could be construed as a potential conflict of interest.

## Publisher's Note

All claims expressed in this article are solely those of the authors and do not necessarily represent those of their affiliated organizations, or those of the publisher, the editors and the reviewers. Any product that may be evaluated in this article, or claim that may be made by its manufacturer, is not guaranteed or endorsed by the publisher.
