# Supplementary material for: The Epidemiology of Lung Metastases
Source: Front Med (Lausanne). 2021 Sep 20;8:723396. doi: 10.3389/fmed.2021.723396 (PMC8488106; doi:10.3389/fmed.2021.723396)
Supplement: Supplementary file 4 [file Table_4.DOCX]

**Supplemental Table 4.** Summary of baseline characteristics for de novo metastatic cases with and without lung metastasis in the United States from 2010 to 2015.

|  | **Lung metastasis** (N = 96,535 [29.0%]) | **No lung metastasis**  (N = 236,875 [71.0%]) | ***P*-value** |
| --- | --- | --- | --- |
| Age, mean (standard deviation) | 66.9 (13.9) | 67.0 (13.1) | 0.017 |
| Sex  Female  Male | 46,773 (47.0%)  49,762 (53.0%) | 111,226 (48.5%)  125,649 (51.5%) | <0.001 |
| Year of diagnosis  2010  2011  2012  2013  2014  2015 | 14,485 (15.0%)  15.267 (15.8%)  16,100 (16.7%)  16,640 (17.2%)  16,996 (17.6%)  17,047 (17.7%) | 38,606 (16.3%)  37,529 (15.8%)  38,875 (16.4%)  39,584 (16.7%)  40,946 (17.3%)  41,335 (17.4%) | <0.001 |
| Presence of bone, brain or liver metastasis at diagnosis  Yes  No | 52,600 (54.5%)  43,935 (45.5%) | 159,234 (67.2%)  77,641 (32.8%) | <0.001 |
| Race  American Indian/Alaska Native  Asian or Pacific Islander  Black  White  Unknown | 753 (0.8%)  7,603 (7.9%)  12,679 (13.1%)  75,299 (78.0%)  201 (0.2%) | 1,447 (0.6%)  16,099 (6.8%)  31,024 (13.1%)  187,713 (79.2%)  592 (0.2%) | <0.001 |
| T-Stage  T0  T1  T2  T3  T4  TX  Other T  Missing | 1,671 (1.7%)  7,962 (8.2%)  13,410 (13.9%)  24,644 (25.5%)  29,852 (30.9%)  18,993 (19.7%)  0 (0%)  3 (<0.1%) | 3,833 (1.6%)  27,342 (11.5%)  48,282 (20.4%)  55,204 (23.3%)  50,239 (21.2%)  51,971 (21.9%)  0 (0%)  4 (<0.1%) | <0.001 |
| N-Stage  N0  N+  NX  Missing | 30,862 (32.0%)  53,501 (55.4%)  12,062 (12.5%)  110 (0.1%) | 83,524 (35.3%)  126,410 (53.4%)  26,931 (11.4%)  10 (<0.1%) | <0.001 |
| M-Stage  M1  Other M  Missing | 96,535 (100%)  0 (0%)*  0 (0%)* | 236,875 (100%)  0 (0%)*  0 (0%)* |  |
| Survival duration missing  No  Yes | 96,535 (100%)  0 (%)* | 236,875 (100%)  0 (%)* |  |
| Median follow-up duration (months, 95% confidence interval) | 33 (32-33) | 33 (33-34) | 0.02 |
| Site   \| Anus, Anal Canal and Anorectum \| \| --- \| \| Biliary Tract \| \| Bone and Soft Tissue \| \| Breast \| \| Cervix Uteri \| \| Colon \| \| Esophagus \| \| Hypopharynx \| \| Kidney and Renal Pelvis \| \| Larynx \| \| Liver \| \| Lung and Bronchus \| \| Melanoma of the Skin \| \| Mesothelioma \| \| Nasopharynx \| \| Oral Cavity excl. Tongue \| \| Oropharynx \| \| Ovary \| \| Pancreas \| \| Penis \| \| Peritoneum, Omentum and Mesentery \| \| Prostate \| \| Rectosigmoid Junction \| \| Rectum \| \| Retroperitoneum \| \| Salivary Gland \| \| Small Intestine \| \| Stomach \| \| Testis \| \| Thyroid \| \| Tongue \| \| Tonsil \| \| Ureter \| \| Urinary Bladder \| \| Uterus \| \| Vulva and Vagina \| | \| 178 \| \| --- \| \| 1,195 \| \| 2,021 \| \| 6,506 \| \| 823 \| \| 6,562 \| \| 2,115 \| \| 153 \| \| 6,989 \| \| 360 \| \| 2,150 \| \| 41,010 \| \| 2,223 \| \| 271 \| \| 137 \| \| 174 \| \| 96 \| \| 1,931 \| \| 6,750 \| \| 41 \| \| 198 \| \| 1,460 \| \| 1,018 \| \| 2,740 \| \| 133 \| \| 219 \| \| 287 \| \| 1,886 \| \| 1,042 \| \| 1,053 \| \| 315 \| \| 172 \| \| 108 \| \| 1,418 \| \| 2,057 \| \| 174 \| | \| 528 \| \| --- \| \| 4,641 \| \| 1,269 \| \| 13,887 \| \| 1,789 \| \| 24,300 \| \| 4,744 \| \| 120 \| \| 4,757 \| \| 204 \| \| 2,901 \| \| 95,598 \| \| 2,273 \| \| 699 \| \| 231 \| \| 164 \| \| 74 \| \| 6,700 \| \| 24,628 \| \| 36 \| \| 804 \| \| 15,781 \| \| 2,482 \| \| 4,945 \| \| 185 \| \| 225 \| \| 2,700 \| \| 10,321 \| \| 655 \| \| 649 \| \| 266 \| \| 211 \| \| 175 \| \| 2,584 \| \| 3,943 \| \| 326 \| | <0.001 |

*P*-values represent results from t-tests, chi-squared tests or log-rank tests for continuous, categorical and time to event variables, respectively, where appropriate. T-Stage, N-Stage and M-Stage represent those from the American Joint Committee on Cancer 7^th^ Edition Staging Manual. *: excluded in the survival analysis.
